# Purification and identification of buckwheat hull flavonoids and its comparative evaluation on antioxidant and cytoprotective activity in vitro

**DOI:** 10.1002/fsn3.1683

**Published:** 2020-06-01

**Authors:** Yang Cui, Ziying Zhao, Ziqi Liu, Junmei Liu, Chunhong Piao, Dailin Liu

**Affiliations:** ^1^ College of Food Science and Engineering Jilin Agricultural University Changchun China; ^2^ Department of Pharmacognosy Logistics University of People's Armed Police Force Tianjin China

**Keywords:** antioxidant activity, buckwheat hulls, cytoprotective activity, flavonoid components, purification and identification

## Abstract

Buckwheat hulls, by‐products of buckwheat processing, contain various flavonoids, but the antioxidant capacity and synergy of different flavonoids are unclear. The purpose of this study was to evaluate the antioxidant activity of flavonoid monomers and high‐flavonoid component (HBHF) in buckwheat hulls using chemical‐based assays and cellular‐based assays. Four kinds of flavonoids were identified as rutin, isoorientin, vitexin, and hyperoside from buckwheat hulls. In chemical‐based assays, rutin and HBHF showed relatively high free radical scavenging activities and total antioxidant capacities. In cellular‐based assays, however, HBHF showed much higher proliferation activity against cell damage than flavonoid monomers when HepG2 cell was oxidatively damaged by H_2_O_2_ or high glucose. The cytoprotective activities of flavonoid monomers and HBHF were closely associated with reducing levels of reactive oxygen species (ROS) and malondialdehyde (MDA) or increasing catalase (CAT) activity. In conclusion, buckwheat hull flavonoids are the favorable application candidates for natural antioxidants.

## Graphical abstract

This work was aimed at the evaluation of antioxidant and cell protective activities of buckwheat hull flavonoids and providing a favorable evidence for the therapeutic utilization of natural extract.

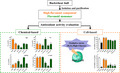

## 1 INTRODUCTION

Flavonoids are a variety of biologically active polyphenolic compounds that are abundant in dietary plants and herbs and possess beneficial effects such as antioxidant, anticancer, anticardiovascular disease, and antidiabetes activities due to the structural diversity of flavonoid subgroups (Maaliki, Shaito, Pintus, El‐Yazbi, & Eid, 2019). Common buckwheat is the most commonly cultivated species around the world (Park, Kim, Lee, Lim, & Hwang, 2019), and large amounts of by‐products, mainly buckwheat hulls, are produced during the process. Numerous studies have shown that more amounts of flavonoids are contained in buckwheat hulls than grains, such as rutin, vitexin, quercetin, isoorientin, and hyperoside (Dziadek et al., 2016; Li, Yuan, Yang, Tao, & Ming, 2013; Sedej et al., 2012; Watanabe, Ohshita, & Tsushida, 1997; Zhang, Zhu, Liu, Bao, & Liu, 2017).

Our previous research suggested that rutin was not considered to be the dominant flavonoid in buckwheat hulls (Zhao et al., 2018). Li et al. (2013), Dziadek et al. (2016), and Zhang et al. (2017) reported that the content of flavonoid compounds in buckwheat hulls differed significantly among the various buckwheat producing areas and cultivars, and our results are relevant to these studies. We also found that buckwheat hull flavonoids that was partly purified had a strong inhibitory effect of *α*‐glycosidase and advanced glycation end products (AGEs) (Zhao et al., 2018) that closely related to the antioxidant activity (Rhee & Kim, 2018), and the therapeutic effects were further confirmed greatly in the model of type 2 diabetes rats and *db*/*db* mice (Li et al., 2017, 2019). From this, we speculated that the flavonoid components have potential therapeutic activities and that the partially purified components may have equal or more effective effects than the monomer components. Therefore, it is necessary to determine the specific chemical composition of therapeutic substances in buckwheat hulls by isolation and purification to explore the therapeutic flavonoid components.

Many studies have reported that antioxidant function has been linked to the treatment of many major diseases such as cancer (Chikara et al., 2018), cardiovascular disease (Siti, Kamisah, & Kamsiah, 2015), and diabetes (Zatalia & Sanusi, 2013). At present, there are various methods for assessing the antioxidant capacity of natural products in vitro and in vivo. Animal experiments and human studies in vivo are more suitable to present the antioxidant activities of samples, but they are not applicable due to time‐consuming and high cost (Xu et al., 2017), and their interference of in vivo tests on physiopharmacological processes such as absorption, distribution, metabolism, storage, and excretion (Kasote, Katyare, Hegde, & Bae, 2015), while in vitro tests, such as free radical scavenging activity assays or the determination of cell viabilities under oxidative stress, are quick and effective. However, the results in measurements based on different methods often differ (Kasote et al., 2015) so that multiple biological assays are necessary for evaluation, including chemical‐based and cellular‐based assays.

In this study, isolation and purification of high‐flavonoid components and identified flavonoid monomers from buckwheat hulls were carried out and their antioxidant activities based on chemical and cellular methods were further investigated. This study provided strong evidence for the exact utilization of buckwheat hulls as a kind of functional food resource, as well as the possibility of using buckwheat hull as a source of low‐cost natural antioxidants.

## 2 MATERIALS AND METHODS

### 2.1 Experimental materials

Buckwheat (*Fagopyrum esculentum* Moench) hulls were purchased from ChiFeng. Rutin, isoorientin, vitexin, and hyperoside (standard ≥98%) were obtained from Nanjing Jingzhu Bio‐technology Co. Ltd., macroporous resin D101 and ADS‐7 were from Dong Hong chemical company, and ODS were from Nacalai Tosoh Inc. Acetonitrile and methanol (chromatographic purity) were supplied by TEDIA. Fetal bovine serum (FBS) was purchased from Biological Industries (BI) Co. Ltd., Dulbecco's modified Eagle medium (DMEM) and glucose solution were from Gibco, and 3‐(4,5‐Dimethylthiazol‐2‐yl)‐2,5‐diphenyltetrazolium bromide (MTT) were from Amresco. All other chemical reagents were of analytic grade from domestic.

### 2.2 Purification and characterization

Preparation of crude buckwheat hull flavonoids according to the methods reported previously (Zhao et al., 2018). Enriched buckwheat hull flavonoids (EBHF) were acquired through column chromatography with macroporous resin D101. Further purification of EBHF was carried out using macroporous resin ADS‐7 with a gradient of ethanol/water (0/100, 0/100, 10/90, 40/60, and 95/5, v/v), and the high content of buckwheat hull flavonoids (HBHF) was obtained with ethanol/water (40/60). Then, HBHF was further eluted stepwise on the ODS column by methanol/water (2/3, 2/3, and 3/2, v/v) and acquired three subfractions of HBHF‐1, HBHF‐2, and HBHF‐3. Compound **1** was crystallized from HBHF‐1 directly, and compound **2** and compound **3** were isolated from HBHF‐2 using PHPLC (LC‐6AD, Shimadzu) with UV‐3000 detector by the mobile phase included solvent A (13% acetonitrile) and solvent B (water/formic acid, 99:1, v/v) in the flow rate of 8 ml/min. All samples were concentrated at 70–80°C using a rotary evaporator (RE52CS, Yarong) and dried by a freeze dryer (FDU‐7006, Operon) and stored at 4°C until use. The partly purified components and flavonoid monomers with different flavonoid content obtained through isolation and purification were collectively referred to as buckwheat hull flavonoids (BHF).

### 2.3 Determination of total flavonoids

The determination of total flavonoids was performed using a colorimetric method described previously (Yi, Yu, Liang, & Zeng, 2008). The results were calculated from a standard curve with concentrations (0.01–0.12 mg/ml) of rutin (linear equation *Y* = 0.2976*X* ＋ 0.0522; *R* ^2^ =.9994) and expressed in mg of rutin equivalent per g of dried sample.

### 2.4 HPLC analysis

The dry samples were dissolved in methanol and passed through a 0.45‐μm microporous membrane filter. LCMS2020 high‐performance liquid chromatography–mass spectrometer (Shimadzu), DAD detector, and a Welchrom C18 (250 mm × 4.6 mm, 5 μm) column were used. The detection wavelength was 280 nm, the column temperature was 30°C, and the injection volume was 10 μl. Acetonitrile and distilled water with 1% formic acid were used as the mobile phase. Elution conditions were 0–10 min (10% acetonitrile), 10–40 min (30% acetonitrile), 40–50 min (50% acetonitrile), 50–55 min (50%–10% acetonitrile), and 55–65 min (10% acetonitrile). The flow rate was set at 1 ml/min.

### 2.5 NMR analysis

Compounds **1**, **2**, and **3**, the monomers which obtained in the isolation and purification, were dissolved in DMSO‐*d*_6_ and analyzed by ^13^C‐NMR and ^1^H‐NMR, using a Bruker ARX‐400 type nuclear magnetic resonance spectrometer (Bruker Co.) operating at 100 MHz. Chemical shifts (δ) were reported in parts per million (ppm), and coupling constants (*J*) were expressed in hertz (Hz). This part of the test data was entrusted to the Shenzhen Chinese Medicine and Natural Medicine Research Center for determination.

### 2.6 Antioxidant activity on chemical‐based assays

Chemical assays are based on the ability to scavenge the stable free radicals, such as hydroxyl radical (•OH) assay, superoxide anion radical ( $O_{2}^{\cdot -}$ ) assay, 1,1‐diphenyl‐2‐picrylhydrazyl (DPPH•) assay, and total antioxidant capacity (T‐AOC).

#### 2.6.1 Free radical scavenging activity assays

Free radical scavenging activity was expressed as IC_50_, defined as the concentration of antioxidant required at a clearance of 50%. IC_50_ was calculated based on the linear regression of the percentage of remaining radical against the concentration of the samples. Particularly, radicals of •OH, $O_{2}^{\cdot -}$ , and DPPH• scavenging activities were evaluated according to the detailed methods (Yi et al., 2008).

$$\text{Scavenging}\;\text{activity}\left(\%\right)=\frac{A_{\text{control}}-A_{\text{sample}}}{A_{\text{control}}}\times 100\%$$

where *A*_control_ was the absorbance of the control reaction (replacing the sample with distilled water), and *A*_sample_ was the absorbance of the sample.

#### 2.6.2 Total antioxidant capacity

Total antioxidant capacity (T‐AOC) was evaluated by the T‐AOC kit (Jiancheng Bioengineering Research Institute) according to the kit instructions. The results were calculated from a standard curve with known concentrations (0.15–1.5 mM) of FeSO_4_‐7H_2_O (linear equation *Y* = 3.7208*X *− 0.3275; *R* ^2^ = 0.9967) and expressed in mmol of FeSO_4_‐7H_2_O equivalent (FE) per g of dried sample.

### 2.7 Antioxidant activity on cellular‐based assays

Cellular‐based assays are to observe whether BHF have protective effects through oxidative damage induced by different inducers (H_2_O_2_ or high glucose) and evaluate cell antioxidant capacity by determination of reactive oxygen species (ROS), malondialdehyde (MDA) levels, and catalase (CAT) activity, to assess the antioxidant capacity of BHF more comprehensively.

#### 2.7.1 Determination of HepG2 cell cytotoxicity

HepG2 cells were provided kindly from the college of life sciences, Jilin Agricultural University. Cells were maintained in DMEM supplemented with 10% FBS, streptomycin (100 µg/ml) and penicillin (100 U/ml) at 37°C in a humidified 5% CO_2_ atmosphere. Cells were seeded at a density of 2 × 10^4^ cells/well in 96‐well microplate. One day after the inoculation, the cells were treated with a medium containing the samples having a concentration of 10, 50, 100, 200, 500, and 1,000 μg/ml, and were cultured for 24 hr. At the end of the incubation, the MTT assay was performed as described to determine cell viability (Liu, Chen, Yang, & Chiang, 2008).

#### 2.7.2 Protective effects of BHF against oxidative stress

HepG2 cells were seeded in 96‐well plates (2 × 10^4^ cells/well) and incubated at 37°C in 5% CO_2_ for 24 hr. The cells were pretreated with samples for 20 hr before exposure to 3.5 mM H_2_O_2_ or 800 mM glucose for a further 4 hr. The cell viabilities were determined using MTT and were expressed as a percentage of the control (Ding et al., 2019; Zou et al., 2018).

#### 2.7.3 Measurement of intracellular ROS

Intracellular ROS was monitored using the DCFH‐DA fluorescence probe assay (Zou et al., 2018). HepG2 cells were seeded on 24‐well plates at 1 × 10^5^ cells/well and incubated at 37°C in 5% CO_2_ for 24 hr. The cells were pretreated with samples for 20 hr before exposure to H_2_O_2_ or high glucose for a further 4 hr. Then, DCFH‐DA was added to the wells at a final concentration of 10 µM and incubation continued for 30 min. The cells were collected in 1.5‐ml centrifuge tubes and washed three times in serum‐free medium and suspended in PBS. Fluorescence intensity was immediately measured with a fluorescent microplate reader (FLUOstar Omega, BMG LABTECH) at an excitation wavelength of 485 nm and an emission wavelength of 525 nm.

#### 2.7.4 Determination of MDA level and CAT activity

HepG2 cells in 96‐well plates (2 × 10^4^ cells/well) were cultured according to the procedures described above. The cell supernatant was removed. The wells were washed three times in PBS and added with 100 µl of 1% Triton X‐100, and then continued to incubate 30 min. The cell lysate was collected and frozen at −80°C for later use. Intracellular MDA level and CAT activity were measured using appropriate assay kits (Jiancheng Bioengineering Research Institute).

### 2.8 Statistical analysis

Each experiment was performed in triplicate or sextuplicate. Data were represented as mean values ± standard deviations (*SD*). The statistical analysis was carried out by using GraphPad Prism 7.0 and Microsoft Excel software. Data were statistically analyzed using SPSS 22 software (SPSS Inc), and differences were deemed significant at *p* <.05.

## 3 RESULTS

### 3.1 Isolation and identification of flavonoid monomers from buckwheat hulls

**FIGURE 1:**
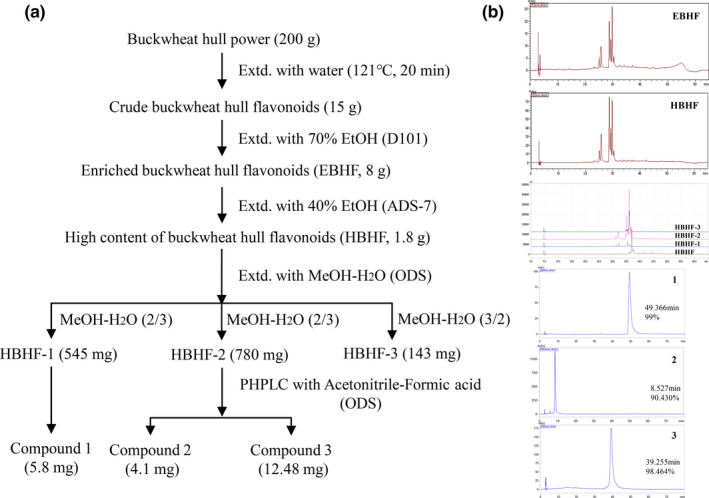
The isolation process and HPLC analysis of BHF from buckwheat hulls. (a) The step of the isolation and purification and (b) HPLC analysis

**FIGURE 2:**
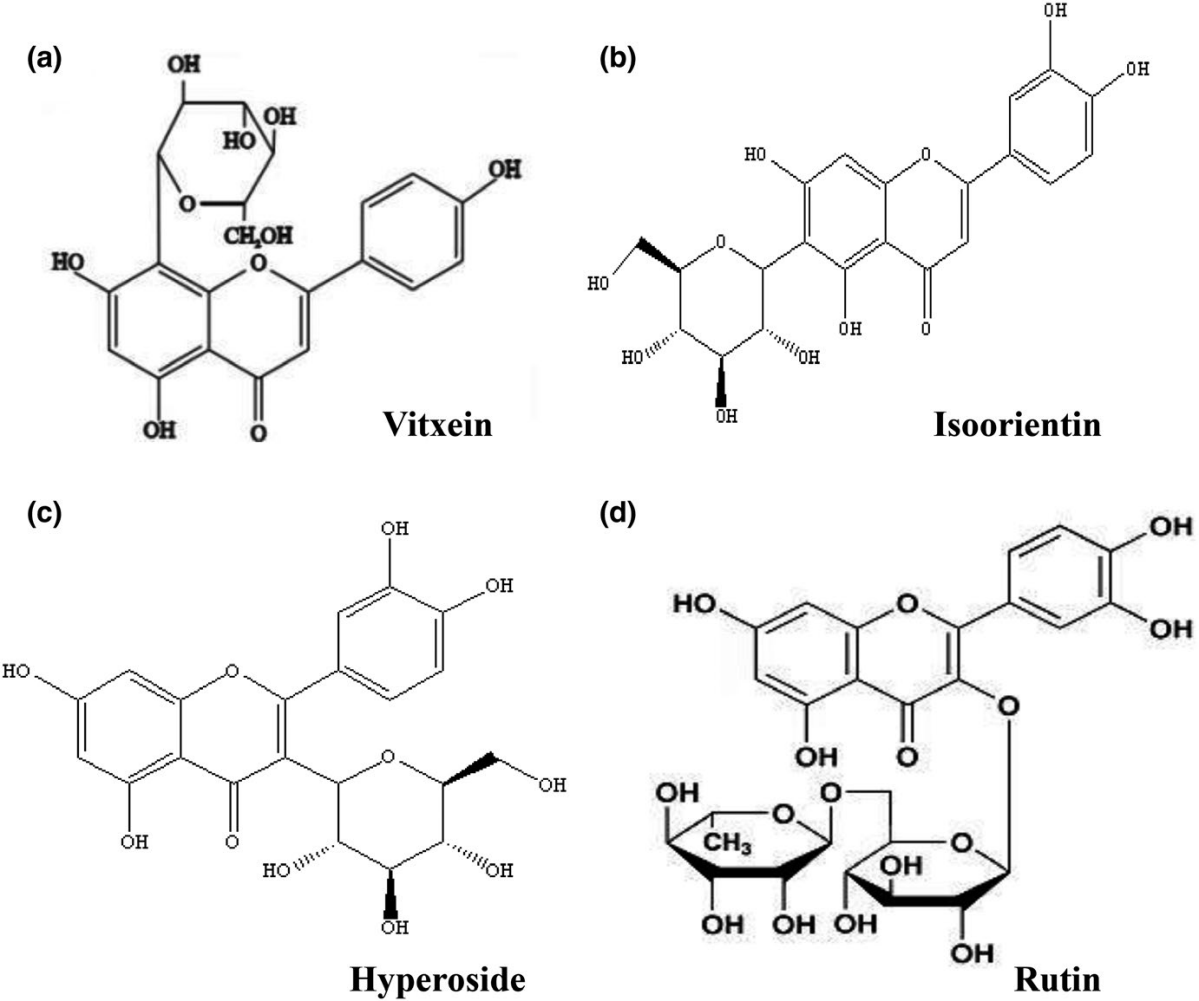
Chemical structures of four kinds of flavonoids. (a) Vitexin; (b) isoorientin; (c) hyperoside; and (d) rutin

Total two kinds of macroporous resin, ODS column chromatography and PHPLC were used for isolation and purification of crude buckwheat hull flavonoids. The isolation process and HPLC analysis are shown in Figure 1. After the first purification with D101, the purity of total flavonoids increased from 308 to 653 mg/g. From the second purification with ADS‐7, the component of HBHF was 892 mg/g total flavonoids and 22.51% yield, which means that the flavonoids were concentrated in HBHF. After further purification, three monomer compounds were separated from HBHF, and the reaction with aluminum trichloride was yellow‐green, and which was positive in the reaction with hydrochloric acid and magnesium powder. Three compounds were determined as flavonoids by spectroscopic analysis with literature data (Datta, Datta, & Sarker, 2000; Guimarães et al., 2015; Liu et al., 2011), and the NMR data are given in Table 1. Compound **1** was identified as vitexin (C_21_H_20_O_10_), a pale yellow needle crystal (methanol), compound **2** as isoorientin (C_21_H_20_O_11_), which was a yellow powder, and compound **3** as hyperoside (C_21_H_20_O_12_), which was brown (methanol). And the chemical structures of compounds **1**, **2**, and **3** are shown in Figure 2. Rutin was not collected and analyzed during the purification in this paper because it was confirmed by HPLC‐MS analysis before.

**TABLE 1:** ^1^H (400 MHz) and ^13^C (100 MHz) NMR spectral data of compounds **1**, **2**, and **3** in DMSO‐*d*_6_ (δ in ppm, *J* in Hz)

Pos.	Compound **1**		Compound **2**		Compound **3**	
	δ_H_	δ_C_	δ_H_	δ_C_	δ_H_	δ_C_
2		163.9		163.3		164.7
3	6.77 (1H,s)	102.5	6.48 (1H,s)	102.8		121.9
4		182.1		181.8		
5		161.3		156.2		156.1
6	6.27 (1H,s)	98.2		108.9	6.18 (1H,d,1.8)	98.8
7		162.5		163.6		161.1
8		104.6	6.69 (1H,s)	93.5	6.89 (1H,d,1.8)	101.9
9		155.9		160.7		156.3
10		103.9		103.4		103.6
C=O						177.3
1′		121.6		121.4		133.4
2′	8.01 (2H,d,8.8)	128.5	7.41 (1H,s)	113.3	7.74 (1H,d,1.8)	115.1
3′	6.88 (2H,d,8.8)	115.9		145.7		148.5
4′		160.3		149.7		144.8
5′	6.88 (2H,d,8.8)	115.9	6.89 (1H,d,8.1)	116.1	6.85 (1H,d,8.4)	115.9
6′	8.01 (2H,d,8.8)	128.9	7.42 (1H,d,8.1)	119.0	7.48 (1H,dd,1.8,8.4)	121.0
1″	4.69 (1H,d)	73.0		73.0	5.35 (1H,d,7.8)	93.5
2″		70.9		70.6		71.2
3″		78.6		78.9		73.2
4″		70.6		70.2		67.9
5″		81.8		81.5		75.8
6″		61.3		61.5		60.1
3′‐OH			9.95 (1H,s)			
4′‐OH	10.30 (1H,s)		9.44 (1H,s)			
5‐OH	13.16 (1H,s)		13.58 (1H,s)		12.57 (1H,s)	
7‐OH	10.79 (1H,s)		10.61 (1H,s)			

### 3.2 Antioxidant activities of BHF on chemical‐based assays

**FIGURE 3:**
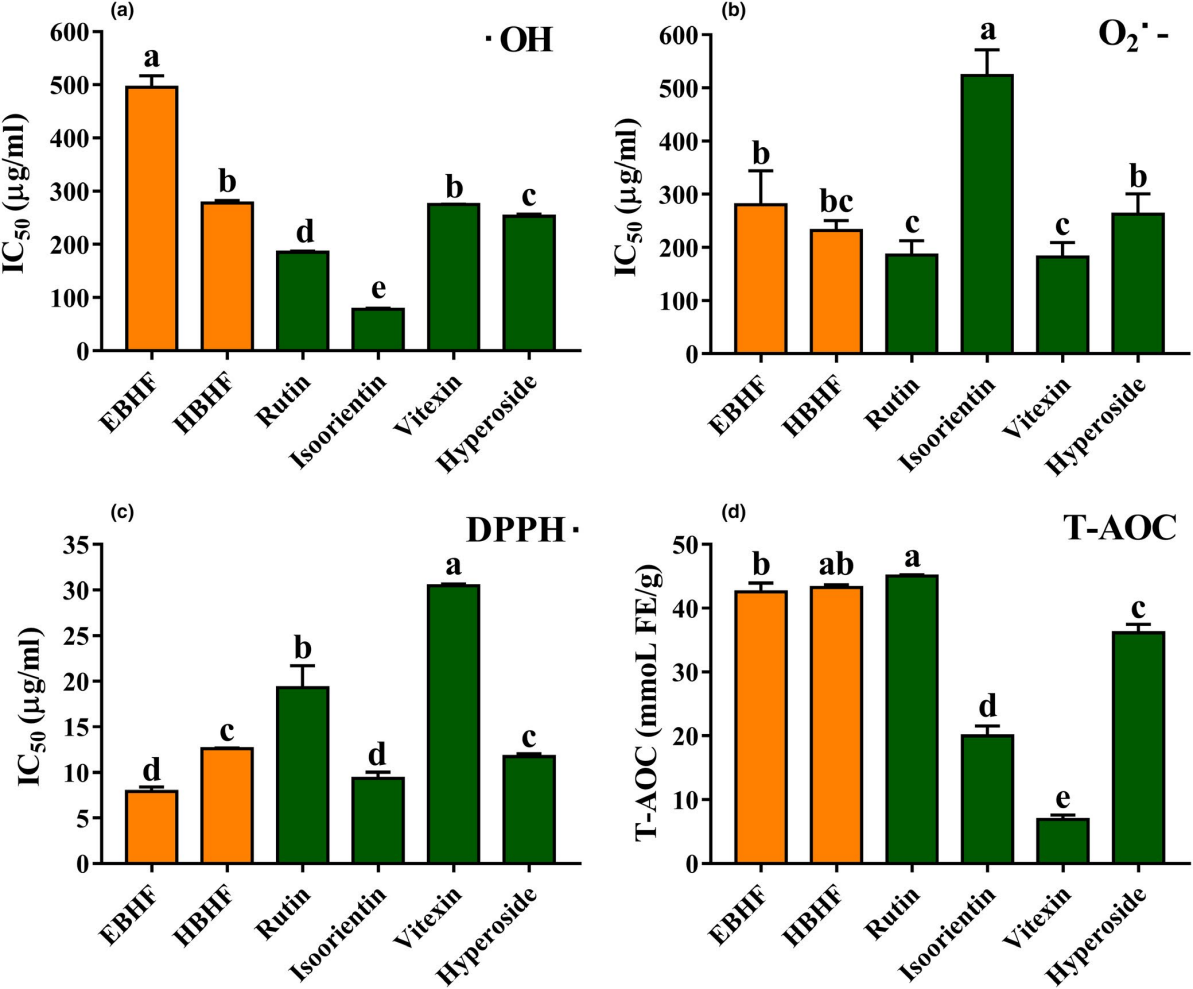
Free radical scavenging activities and total antioxidant capacity of BHF from buckwheat hulls. (a) •OH radical scavenging activity; (b) $O_{2}^{\cdot -}$ radical scavenging activity; (c) DPPH• radical scavenging activity; and (d) T‐AOC. Values are means ± *SD* (*n* = 3). Different letters (a–e) indicate significant differences among the different samples (*p* <.05, one‐way ANOVA test)

The results of the scavenging •OH, $O_{2}^{\cdot -}$ , DPPH• abilities, and T‐AOC of BHF are summarized in Figure 3. From all radical scavenging activities, HBHF and rutin were exhibited relatively high patterns of antioxidant activity than other flavonoid components (Figure 3a–c). The extent of the inconsistencies activities was observed in four kinds of flavonoid monomers. For example, isoorientin showed the highest •OH (IC_50_ 78.23 ± 1.56 μg/ml) and DPPH• (IC_50_ 9.34 ± 0.55 μg/ml) scavenging activities (Figure 3a,c), while it showed the lowest $O_{2}^{\cdot -}$ (IC_50_ 523.24 ± 39.56 μg/ml) scavenging activity (Figure 3b). One notable fact was that EBHF and HBHF showed much higher of T‐AOC as well as rutin and hyperoside (Figure 3d). It can be concluded that HBHF and rutin in buckwheat hulls had higher antioxidant activities (*p* < .05) and these results predict that HBHF has great potential to be used as a natural antioxidant.

### 3.3 Antioxidant activities of BHF on cellular‐based assays

#### 3.3.1 HepG2 cell cytotoxicity of BHF

**FIGURE 4:**
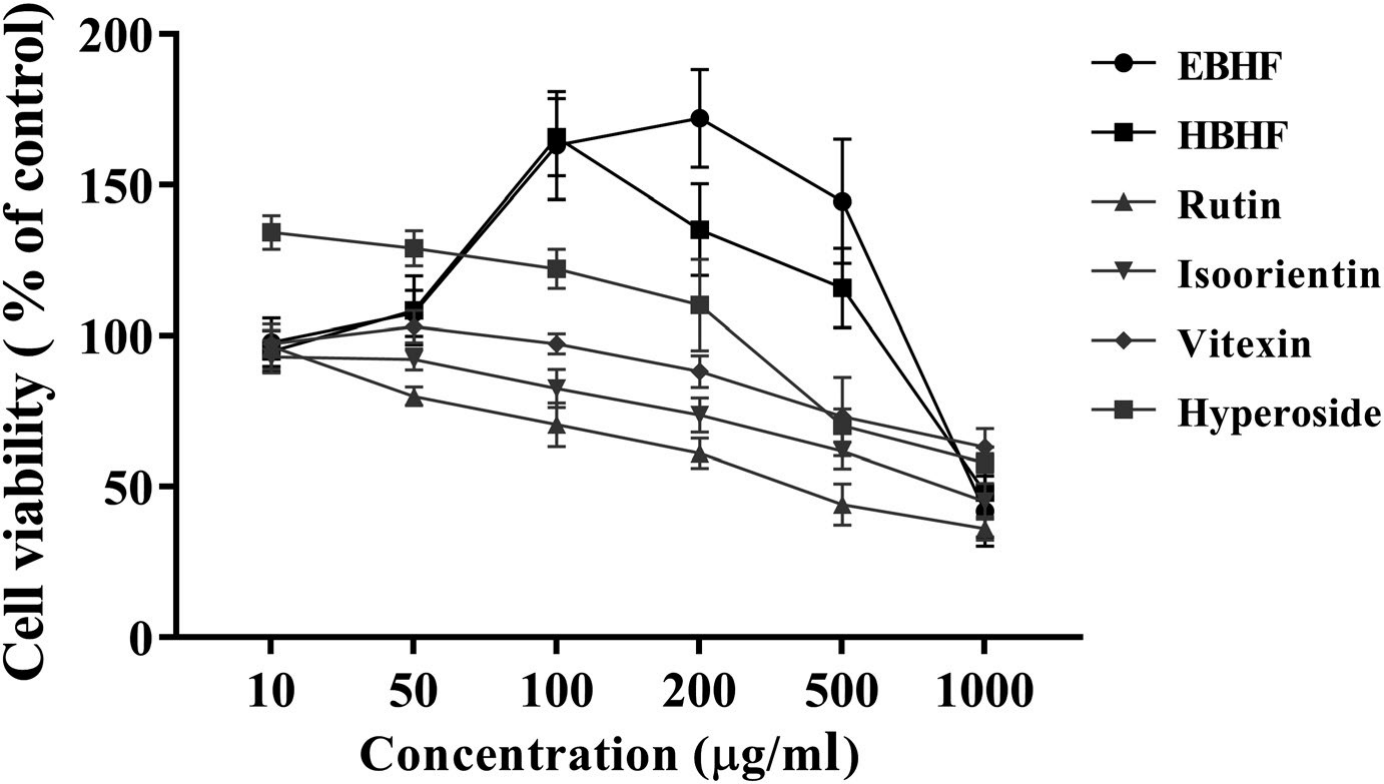
HepG2 cell cytotoxicity of BHF from buckwheat hulls. Cells were treated with the concentrations from 10 to 1,000 μg/ml for 24 hr, and cell viabilities were measured by MTT assay. Data are expressed as means ± *SD* (*n* = 6)

The cytotoxic effects of BHF on HepG2 cells are shown in Figure 4. EBHF and HBHF ranging from 10 to 500 µg/ml had no significant cytotoxicity but increased cell proliferation at a concentration of 50–100 µg/ml. However, all flavonoid monomers, rutin, isoorientin, vitexin, and hyperoside, showed a decreasing trend of the cell viabilities with concentration‐dependent. A lower concentration of 50 μg/ml BHF was selected as the intervention to investigate the amelioration effects on oxidative stress.

#### 3.3.2 Protective activity of BHF from H_2_O_2_‐induced oxidative stress

**FIGURE 5:**
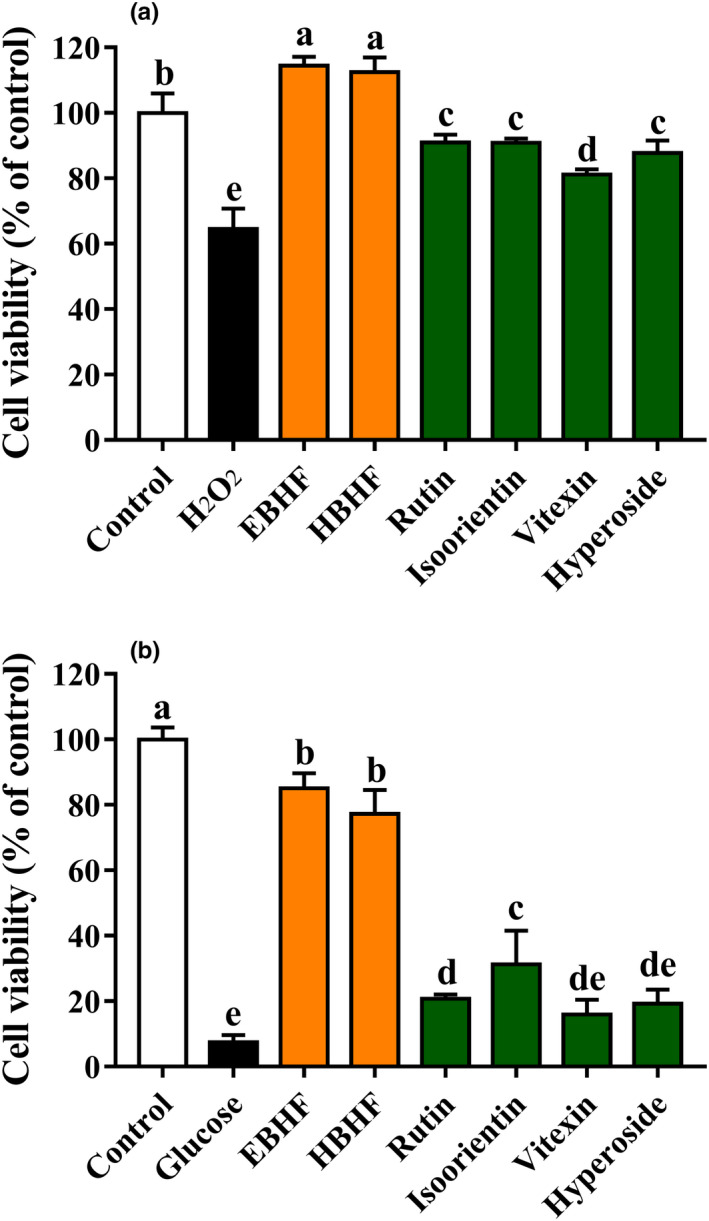
Protective effects of BHF on oxidative stress in HepG2 cells. (a) H_2_O_2_‐induced oxidative stress and (b) high glucose‐induced oxidative stress. Values are means ± *SD* (*n* = 6). Different letters (a–e) indicate significant differences among the different samples (*p* <.05, one‐way ANOVA test)

When HepG2 cells were damaged by H_2_O_2_ stress, the cell viabilities were decreased to 64.55 ± 5.69%, but the cell viabilities were significantly recovered about range from 112.5% to 114.5% after preincubation with EBHF and HBHF, which indicate EBHF and HBHF can improve cell oxidative damage (*p* < .05). The same trend of protective activities was also observed from rutin, isoorientin, vitexin, and hyperoside (81.16%–90.91%), but the protective activities were significantly lower than EBHF and HBHF (Figure 5a).

#### 3.3.3 Protective activity of BHF from high glucose‐induced oxidative stress

The protect activity of HepG2 cells against high glucose‐induced oxidative stress was also determined (Figure 5b). After pretreatment with EBHF and HBHF, all cell viabilities were increased significantly (*p* < .05) with about 80%, while the cell viability was just for 7.41 ± 2.06% under the condition of high glucose‐damaged cells. By contrast, the protective activities of rutin, isoorientin, vitexin, and hyperoside (15.99%–31.21%) were lower than EBHF and HBHF. These data demonstrated that EBHF and HBHF possessed remarkable HepG2 cell protective capacities (Figure 5).

#### 3.3.4 Intracellular ROS, MDA levels, and CAT activity in HepG2 cells

**FIGURE 6:**
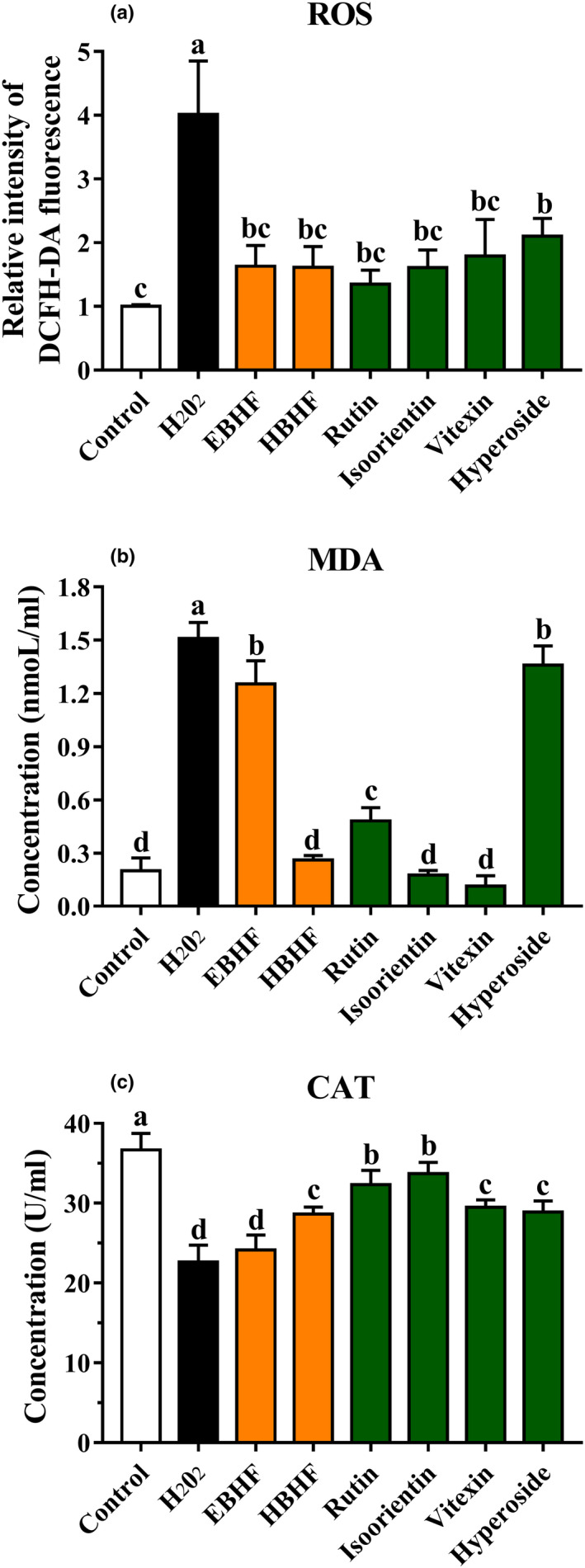
Effects of BHF on oxidative stress factors in HepG2 cells induced by H_2_O_2_. (a) ROS level; (b) MDA level; and (c) CAT activity. ROS levels are expressed by DCFH‐DA fluorescence intensity. The level of DCFH‐DA fluorescence in the control group was designated as 1 and was used to express the relative fluorescence intensity in the other groups. Values are means ± *SD* (*n* = 3). Different letters (a–d) indicate significant differences among the different samples (*p* <.05, one‐way ANOVA test)

**FIGURE 7:**
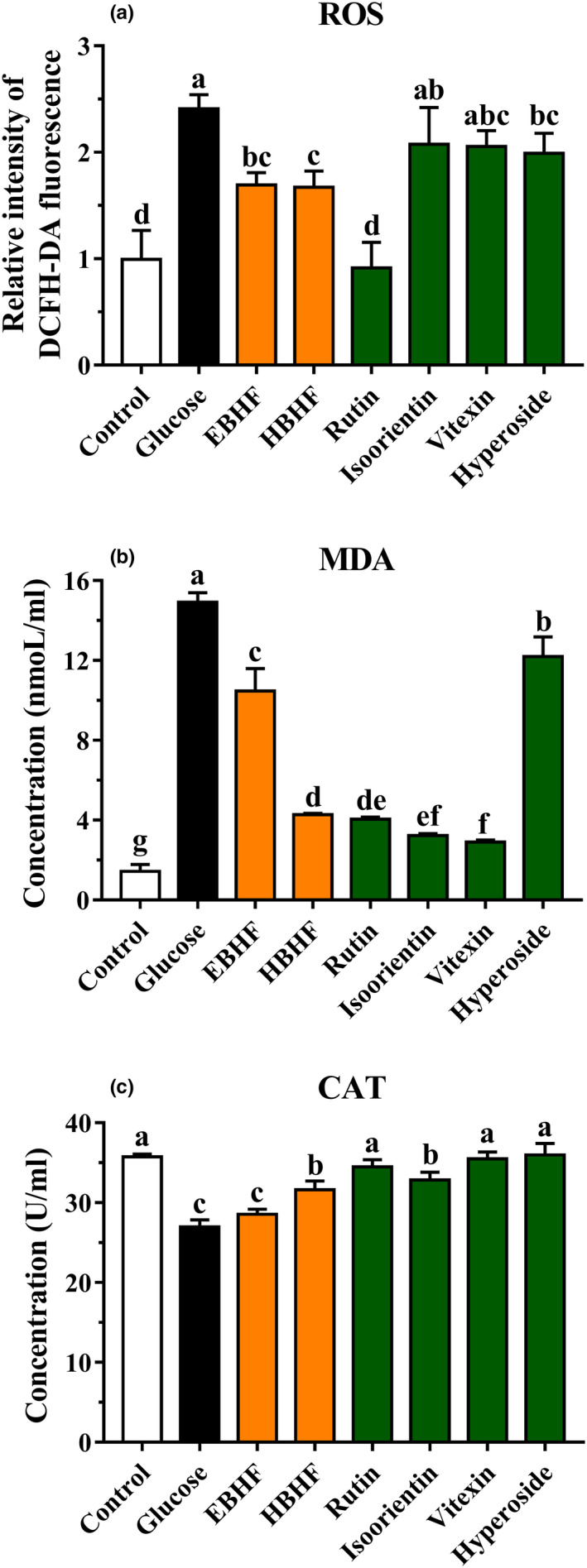
Effects of BHF on oxidative stress factors in HepG2 cells induced by high glucose. (a) ROS level; (b) MDA level; and (c) CAT activity. ROS levels are expressed by DCFH‐DA fluorescence intensity. The level of DCFH‐DA fluorescence in the control group was designated as 1 and was used to express the relative fluorescence intensity in the other groups. Values are means ± *SD* (*n* = 3). Different letters (a–g) indicate significant differences among the different samples (*p* <.05, one‐way ANOVA test)

To evaluate whether the cytoprotective activity was related to antioxidant activity, we also measured intracellular ROS, MDA levels, and CAT activity. When the HepG2 cells were damaged by H_2_O_2_ or high glucose‐induced oxidative stress, high ROS and MDA levels and low CAT activity were observed in the H_2_O_2_ or high glucose group compared with the control group (Figures 6 and 7). These values were significantly ameliorated by pretreatment of HBHF or flavonoid monomers (*p* <.05).

In H_2_O_2_‐induced oxidative damage, there was no significant difference between HBHF and flavonoid monomers in reducing intracellular ROS production, although both of them significantly reduced ROS level (*p* <.05) (Figure 6a). HBHF, rutin, isoorientin, and vitexin significantly reduced the production of MDA (*p* <.05) (Figure 6b). HBHF, rutin, and isoorientin showed a significant increase in CAT activity (*p* <.05) (Figure 6c). Instead, rutin exhibited the greatest ability to reduce ROS level (*p* <.05) in high glucose‐induced oxidative damage (Figure 7a), while HBHF, rutin, isoorientin, and vitexin had an excellent capacity of MDA reduction (*p* <.05) (Figure 7b). HBHF and four kinds of flavonoid monomers raised significant CAT activities (*p* <.05) (Figure 7c). Despite no significant trend was observed in HBHF and flavonoid monomers, they definitely improved cell damage at the level of oxidative stress factors. This may be due to the different types of flavonoid components, which have different mechanisms to improve cell viability.

## 4 DISCUSSION

This study showed that four kinds of flavonoid monomers, rutin, isoorientin, vitexin, and hyperoside, were obtained from buckwheat hulls through isolation and purification, which have also been confirmed in previous studies (Sedej et al., 2012; Watanabe et al., 1997; Zhang et al., 2017). Many studies reported that the content of different flavonoid monomers differed in different origins and species of buckwheat (Dziadek et al., 2016; Li et al., 2013; Zhang et al., 2017). Kreft et al. (2020) showed that the content of flavonoids in buckwheat could be increased through gene breeding, so as to improve the nutritional value of buckwheat. Zhang et al. (2017) reported that seven kinds of orientin, isoorientin, vitexin, isovitexin, hyperoside, rutin, and quercetin were identified from buckwheat hulls, indicating that more buckwheat hull flavonoids need further isolation and identification. Although quercetin is an important active flavonoid in buckwheat, which has been also reported to be detected in the hulls (Sedej et al., 2012; Watanabe et al., 1997; Zhang et al., 2017), it has not been detected in our study. Lee et al. (2016) reported that quercetin was only detected in tartary buckwheat and not in common buckwheat, which is consistent with our results.

The results of antioxidant activity showed that rutin, isoorientin, vitexin, and hyperoside exhibited different scavenging activities against different kinds of free radicals and only rutin showed excellent T‐AOC (Figure 3). Rutin has been known to be the representative flavonoid in buckwheat hulls (Lee et al., 2016; Sedej et al., 2012; Watanabe et al., 1997; Zhang et al., 2017) and rutin had a remarkable antioxidant capacity in chemical‐based assays (Figure 3) but it seemed not so effective with the damageable cell model induced by H_2_O_2_ and high glucose in cellular‐based assays (Figure 5). Isoorientin, vitexin, and hyperoside also showed similar patterns. This effect may be related to poor water solubility (Sun, Zhang, Lu, Zhang, & Zhang, 2011) and a strong cytotoxic effect of flavonoid monomers (Kreft, 2016). Interestingly, HBHF had a similar or higher antioxidant capacity than rutin in chemical‐based assays and also exhibited a higher therapeutic effect in cytoprotective activity to ameliorate H_2_O_2_ and high glucose‐induced cell damage in cellular‐based assays (Figure 5). Therefore, we speculated that there may be a strong synergistic effect among the individual components of HBHF. The therapeutic effect of the components was superior to flavonoid monomers isolated and purified from extract material, which was consistent with the development trend of functional food. Many studies have further proved that the antioxidant capacity of monomer compounds from fresh apple, *Gingko biloba* and *Trollius chinensis* flowers, was not necessarily higher than that of the components before purification (Eberhardt, Lee, & Liu, 2000; Sun et al., 2013; Zhang et al., 2010). At the same time, the buckwheat hull products after in vitro digestion in an artificial gastrointestinal tract had a significant inhibitory effect on the proliferation of HT‐29 cancer cells (Dziedzic et al., 2018), and the buckwheat hull extracts can be used to prolong shelf life of products by protecting them against lipid oxidation and deterioration of their nutritional quality (Hęś et al., 2017). Although the established definitive pharmacodynamics and well‐defined metabolic pathways of monomeric substances are helpful for its mechanism studies (Hu & Xu, 2009; Xu et al., 2017), the high cost for isolation and purification to the substance monomer might limit the application of functional food.

H_2_O_2_ and high glucose can induce significant oxidative stress in cells, which has been widely used to study the ability of many kinds of bioactive material to inhibit oxidative stress (Ding et al., 2019; Zou et al., 2018), resulting in excessive production of ROS. MDA is an end product of lipid peroxidation, and its content is used to estimate the degree of oxidative damage in cells. CAT activity is also a key indicator of antioxidant capacity (Ding et al., 2019; Sun et al., 2011; Zou et al., 2018). The results showed that the cytoprotective activities of HBHF and monomeric flavonoids were closely associated with reducing the levels of ROS and MDA or increasing CAT activity (Figures 6 and 7), which is consistent with the results of Sun et al. (2011). Furthermore, the flavonoid monomers and HBHF had no regular changes in the expression level of oxidative stress factors, and it was speculated that they had unique mechanisms against oxidative stress.

Another very noteworthy thing is that components of BHF greatly improved insulin resistance in the model of type 2 diabetic and had a great inhibitory effect against *α*‐glycosidase and AGEs formation in our previous studies (Li et al., 2017, 2019; Zhao et al., 2018). Therefore, combined with the results of this study, we clearly suggest that the partly purified component of HBHF from buckwheat hull extract is more promising than the monomeric flavonoids because the material has a strong synergistic effect among the individual components of HBHF. Therefore, purification to some extent is very interesting, and the synergy between their compounds must be further studied.

## 5 CONCLUSION

Totally four kinds of flavonoids, rutin, vitexin, isoorientin, and hyperoside, were purified and identified from buckwheat hulls in the study. Compared to the flavonoid monomers, the partly purified component of HBHF had a strong ameliorative effect on cell damage of HepG2 cells induced by H_2_O_2_ and high glucose, while there was no significant difference among them in antioxidant activity evaluated by chemical methods. In summary, our study demonstrated that buckwheat hull flavonoids had a potential therapeutic effect, which provides very supportive evidence for the extensive utilization of natural extracts.

## CONFLICT OF INTEREST

All authors declare that they have no conflicts of interest.
